# The Human Male Liver Is Predisposed to Inflammation *Via* Enhanced Myeloid Responses to Inflammatory Triggers

**DOI:** 10.3389/fimmu.2022.818612

**Published:** 2022-04-14

**Authors:** Adrian Kuipery, Deeqa Mahamed, Shirin Nkongolo, June Ann D’Angelo, Alexandra Johnson Valiente, Aman Mehrotra, William C. Chapman, Peter Horton, Ian McGilvray, Harry L. A. Janssen, Adam J. Gehring

**Affiliations:** ^1^ Toronto Centre for Liver Disease, Toronto General Hospital Research Institute, University Health Network, Toronto, ON, Canada; ^2^ Department of Immunology, University of Toronto, Toronto, ON, Canada; ^3^ Molecular Microbiology and Immunology, Saint Louis University School of Medicine, St. Louis, MO, United States; ^4^ Division of Abdominal Transplant, Washington University School of Medicine, St. Louis, MO, United States; ^5^ Methodist University Hospital Transplant Institute, Memphis, TN, United States; ^6^ Division of Abdominal Transplant, Saint Louis University School of Medicine, St. Louis, MO, United States; ^7^ The University of Tennessee Health Science Center, Memphis, TN, United States; ^8^ Multi-Organ Transplant Program, Toronto General Hospital Research Institute, Toronto, ON, Canada

**Keywords:** sex, liver, monocytes, Hepatitis B virus, TLR signaling

## Abstract

**Background & Aim:**

Men have a higher prevalence of liver disease. Liver myeloid cells can regulate tissue inflammation, which drives progression of liver disease. We hypothesized that sex alters the responsiveness of liver myeloid cells, predisposing men to severe liver inflammation.

**Methods:**

Luminex was done on plasma from Hepatitis B Virus infected patients undergoing nucleoside analogue cessation in 45 male and female patients. We collected immune cells from the sinusoids of uninfected livers of 53 male and female donors. Multiparametric flow cytometry was used to phenotype and characterize immune composition. Isolated monocytes were stimulated with TLR ligands to measure the inflammatory potential and the expression of regulators of TLR signaling.

**Results:**

We confirmed that men experienced more frequent and severe liver damage upon Hepatitis B Virus reactivation, which was associated with inflammatory markers of myeloid activation. No differences were observed in the frequency or phenotype of sinusoidal myeloid cells between male and female livers. However, monocytes from male livers produced more inflammatory cytokines and chemokines in response to TLR stimulation than female monocytes. We investigated negative regulators of TLR signaling and found that TOLLIP was elevated in female liver-derived monocytes

**Conclusions:**

Our data show that enhanced responsiveness of myeloid cells from the male liver predisposes men to inflammation, which was associated with altered expression of negative regulators of TLR signaling.

## Lay Summary

800 million people are at risk of developing cirrhosis as a result of chronic liver disease and this can put people at higher risk for liver cancer. The progression of chronic liver disease is caused by altered regulation of the immune response in the liver by viral infection, alcoholism or obesity. These external factors cause over-activation of immune cells in the liver, which kills liver cells, hepatocytes, which are then replaced by scar tissue. Epidemiological data clearly indicates that men are more likely to develop cirrhosis and liver cancer. We hypothesized that immune cells in the liver of men display enhanced activation to environmental triggers like viral infection, compared to women. We show here that specific immune cells in the liver, monocytes, look similar between men and women but show potential for enhanced inflammation upon activation. This enhanced inflammation could explain increased risk for men to develop cirrhosis and liver cancer.

## Introduction

The liver is central to the overall health of the individual. The physiological function of the hepatocyte containing parenchyma contributes to nutrient transport, energy, fat storage, digestion, clotting factor production, and detoxification (1). However, the liver is also considered an immunological organ (2). The entire blood volume flows through the liver every 5 min, with 80% of that blood derived from the portal venous system (1). Portal blood is laden with bacterial and food antigens that are removed by immune cells lining the unique capillary bed of the liver. This system, when working properly, removes potentially toxic substances with minimal inflammation or damage to the normal physiological function. However, when dysregulated, the intrahepatic immune system causes chronic inflammatory disease that leads to fibrosis and cirrhosis, potentially ending in liver cancer (3).

Recent estimates put more than 800 million people at risk for the development of cirrhosis as a result of chronic liver inflammation (4). Epidemiological data highlights a clear disparity, based on sex, that underlies progression of liver disease. Men are more likely to develop cirrhosis than women (5), translating to the fact that men are three times more likely to develop liver cancer than women (6). Overall, men are more likely to die from chronic liver disease than women (7). Given the different outcomes of liver disease in men, compared to women, we hypothesized that the intrahepatic immune system would display differences in either composition or function between men and women.

Under normal conditions, the liver displays a tolerogenic environment that suppresses immune activation in the tissue (8). This environmental regulation is primarily controlled by liver and sinusoid resident myeloid cells, including macrophages and monocytes. Liver macrophages, or Kupffer cells, are a heterogeneous population of macrophages that produce IL-10 and TGF-β in response to LPS to maintain the suppressive liver environment (9). However, exposure to pathogen-associated molecular patterns (PAMPs) breaks the tolerogenic environment, triggering an inflammatory cascade that recruits inflammatory immune cells to the liver, causing non-specific hepatocyte killing (10). Resident within the liver and sinusoids, and recruited early in the inflammatory process, is a significant monocyte population with the plasticity to develop into inflammatory macrophages (11, 12). Given the central role of myeloid cells in the inflammatory process, we hypothesized the myeloid population within the liver may be an important source of the sexual dimorphism observed in the progression of chronic liver inflammation.

To investigate this hypothesis, we first validated the male-bias for hepatitis in a clinical cohort of chronic hepatitis B (CHB) patients that developed liver inflammation due to Hepatitis B virus (HBV) reactivation. Subsequently, we analyzed the composition and functional profile of immune cells isolated from uninfected donor livers prior to transplant. Our data indicate that biological sex did not significantly alter the composition or phenotype of myeloid cells derived from the human liver. However, exposure to Toll-like receptor (TLR) ligands unmasked a significant sex-bias in the inflammatory potential of intrahepatic monocytes. Finally, we suggest that males are at a higher risk for the development of liver disease due to lower expression of negative regulators of NF-ĸB signaling. Our data provides evidence in the human liver to explain male-biased progression of inflammatory liver disease.

## Materials and Methods

### Ethics Statement

Peripheral blood and liver perfusion samples were collected from Saint Louis University Hospital and Barnes Jewish Hospital, in collaboration with Mid-America Transplant Services in Saint Louis, Missouri. Perfusion samples were also collected at the Toronto General Hospital, Toronto, Canada. Informed, written consent was obtained from next of kin. The study was approved by the Saint Louis University institutional review board and the University Health Network research ethics board.

### Plasma Samples From Chronic Hepatitis B Patients

Plasma was collected from 45 patients who underwent a protocol-defined stop to their antiviral therapy for chronic HBV infection. The clinical characteristics of the patients and outcome of the study was previously published (13). Patient plasma was analyzed using a custom Luminex Bead Assay (R&D Systems) to measure CD14, soluble CD163 (sCD163), IL-1α, IL-1β, IL-6, TNF-α, FAS-L, TRAIL, and CXCL10 in serum of patients at baseline, 12 w and 18 w after stopping therapy. Each patient’s baseline measurements were subtracted from the 12w and 18w samples to normalize change from baseline.

### Blood Collection and Processing

Blood was collected by standard venipuncture. PBMC were collected by density gradient centrifugation and cryopreserved in liquid nitrogen in 90% knockout serum replacement (KO; Life Tech) + 10% DMSO (Sigma).

### Collection and Characterization of Sinusoidal Resident Intrahepatic Mononuclear Cells

Non-adherent sinusoidal resident intrahepatic mononuclear cells (IHMC) were isolated from the livers of cadaveric donors. Liver donor characteristics can be found in **Supplementary Table 1** and the distribution of cause of death in **Supplementary Table 2**. Peripheral blood and liver perfusion samples were collected at the Mid-America Transplant Services for organs assigned for transplant at the Saint Louis University Hospital or Barnes-Jewish Hospital Abdominal Transplant Programs. Samples were also collected at the Toronto General Hospital. After blood collection, peripheral blood was removed from the liver by aortic flush and IHMC were collected by liver-specific, back table, portal vein perfusion with 1L of University of Wisconsin solution. Mononuclear cells were concentrated using centrifugation at 300xg for 10 min before being layered over ficoll (GE healthcare) to remove residual red blood cell contamination. Following density gradient separation, cells were cryopreserved as above.

### Flow Cytometry

IHMC (1x10^6^ cells) were stained with fixable viability dyes (ThermoFisher Scientific) and fluorochrome conjugated antibodies listed in **Supplementary Table 3**. Samples were acquired on BD flow cytometry instruments and analyzed with FlowJo V10 software (TreeStar Inc). Live singlet monocytes (CD14+CD3-CD7-CD19-CD20-CD56) were down-sampled to 5000 per sample and concatenated before tSNE dimensionality reduction analysis in FlowJo using the following markers for monocytes: CD32, CD64, HLA-DR, CD11b, CD11c, CD35, CD163, CD68, and CD88

### Stimulation of IHMC With TLR Ligands

IHMC monocytes were purified using CD14+ Microbeads (Miltenyi) and cultured for 24h in the presence of TLR ligands (TLR2: 10^8^/ml heat-killed *Listeria monocytogenes*, TLR3: 10 µg/ml polyI:C, TLR4: 1 µg/ml *E. coli* LPS, TLR5: 1 µg/ml *B. subtilis* flagellin, TLR8: 5 µg/ml ssRNA40; Invivogen). Supernatants were collected and measured for the accumulation of 12 different cytokines and chemokines using Cytometric Bead Arrays (BD Biosciences).

### Sex Hormone Exposure and TLR Expression on Monocytes

Total peripheral blood mononuclear cells from 3 healthy donors were cultured in serum free, phenol red-free RPMI 1640 supplemented with 100 U/ml penicillin, 100 µg/ml streptomycin, non-essential amino acids, and 1mM sodium pyruvate. Cells were treated with 1 μM beta-estradiol (EST) or 5a-Dihydrotestosterone (DHT) for 18h and then stimulated with R848 (1 µg/ml) or TL8-506 (TLR8 agonist, 0.1 µg/ml) for 6h in the presence of 10 µg/ml Brefeldin A. Following the stimulation, PBMC were stained with viability dye and stained for CD14. Cells were permeabilized with cytofix/cytoperm (BD Biosciences) and stained for IL-6 or CCL4 for 30 min at 4°C, washed and fixed in 1% paraformaldehyde. Samples were acquired and analyzed using flow cytometry as described above.

To measure TLR expression, IHMC samples from 5 male and 5 female liver donors were stained for viability, CD14, and TLR5, followed by intracellular TLR8 staining in the method described above. Samples were acquired and analyzed using flow cytometry as described above.

### Quantitative PCR of the TLR Pathway

IHMC monocytes from 6 patients were isolated as described above. Following isolation, RNA was isolated using a RNeasy micro kit (Qiagen) and quantified using a NanoDrop 2000. 350 ng of RNA was reverse transcribed using a High-Capacity cDNA Reverse Transcription Kit (Applied Biosystems). Gene expression was measured with pre-designed TaqMan Gene Expression Assay (FAM) kits (Thermo Fisher). TaqMan qPCR was performed using TaqMan Fast Advanced Master Mix (Applied Biosystems) on a QuantStudio 6 thermocycler (Applied Biosystems) to investigate the expression of TLRs and regulators of TLR and expression was normalized to the expression of beta-2-microglobulin

### TOLLIP Western Blot

CD14 monocytes from 10 PBMC and 12 IHMC donors were isolated as described above (PBMC: n = 5 male, 5 female; IHMC: n = 4 male, 8 female). Cells were lysed with RIPA Buffer with Protease Inhibitors (Thermo Scientific) according to manufacturer description. Cell lysate was run on a 12% reducing acrylamide gel and transferred to a PVDF membrane. The membrane was blocked for 90 minutes with MilliQ water with 5% skim milk. TOLLIP (Biolegend) and actin (BD Biosciences) staining was done 1:1000 and 1:10,000, respectively, in TBST with 2% BSA for 1 hour. Secondary staining with HRP conjugated anti-murine IgG at 1:2000 (Biolegend) in TBST with 2% skim milk was done for 1 hour. TOLLIP and actin staining was measured using ECL detection reagents (Bio-Rad) and imaged with a ChemiDoc Imaging System (Bio-Rad).

### Data Analysis and Statistics

Statistical analysis was performed with GraphPad Prism 8 and R V3.5.3. T-sne analysis was performed in FlowJo (parameters: flt-tsne implementation, max iterations 2000, Theta 0.5, learning rate 200). The statistical analysis performed for each experiment is included in the figure legend. Chemiluminescence was measured using Image Lab V6.1.0 (Bio-Rad). Outlier analysis was performed using the Grubb’s method (alpha = 0.05).

## Results

### Enhanced Myeloid Inflammatory Responses in Men After HBV Reactivation

To validate clinical observations that males have worsened disease outcomes in the context of hepatitis, and to identify inflammatory cytokine profiles, we chose to investigate inflammatory cytokine production in males and females following HBV reactivation during nucleoside analogue cessation. HBV reactivation following nucleoside analogue cessation induces rapid rebound in HBV replication and induces liver inflammation and damage in a majority of chronic hepatitis B (CHB) patients (13, 14) However, HBV exclusively replicates within hepatocytes and naturally derived virus does not trigger inflammatory responses *in vivo* (15). The trigger for inflammation is currently unknown but HBV reactivation provides a predictable time frame to compare naturally induced, liver-specific inflammation between men and women.

**Figure 1:**
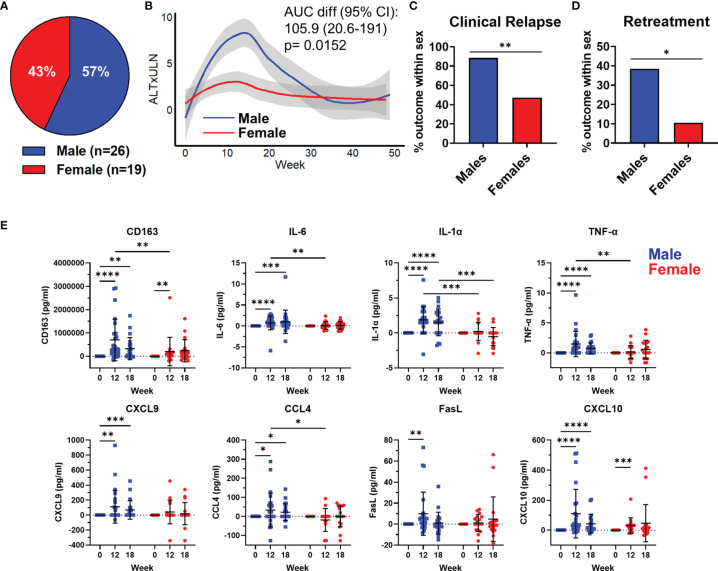
HBV reactivation following treatment cessation induces a myeloid inflammatory profile in the plasma of chronic hepatitis B patients. A description of the cohort has been previously published (13). **(A)** Distribution of participants sex (n=26 males, 19 female) in the Toronto STOP study. **(B)** Loess regression curves of male and female ALT levels as a multiple of upper limit of normal (ALTxULN) during the 48 week follow up to stopping Nuc therapy. **(C)** Incidence of clinical relapse in males and females. **(D)** Proportion of male and female patients who required retreatment after stopping Nuc therapy. **(E)** Plasma levels of cytokines and chemokines at 12w and 18w after stopping Nuc therapy relative to baseline values at week 0. *p<0.05, **p<0.01, ***p<0.001, ****p<0.0001, Mann-Whitney U tests. Figures depict mean +/- SD.

We analyzed the kinetics, magnitude, and plasma immunological profile in 45 chronic hepatitis B (CHB) patients stopping antiviral therapy (13). The participants in this study were 43% female (n=19) and 57% male (n=26; **Figure 1A**). Patients were stratified by sex and the median ALT levels were plotted over a 48w follow-up period. ALT values were normalized for baseline differences between men and women, 25 U/L for females and 35 U/L for males and displayed as the upper limit of normal (ULN) (**Figure 1B**). The peak ALT for men was significantly higher than women (area under the curve (AUC) ± standard error of the mean; males: 240.6 ± 37.8, females: 134.7 ± 21.3), indicative of greater liver damage (**Figure 1B**). We then calculated the frequency of men or women who developed a clinical relapse from HBV reactivation (ALT>1.5xULN and HBV DNA > 2000 IU/ml). We found that 88% of men experienced clinical relapse, whereas only 47% of women relapsed (**Figure 1C**; relative risk of 1.9 (95% CI 1.23-3.28); p=0.0063). This translated clinically into more men having to restart antiviral therapy than women (**Figure 1D**; relative risk for retreatment for males = 3.65 (95% CI 1.07-13.98), p=0.047). These clinical data demonstrate that the male liver is primed for enhanced inflammatory responses with a pathological stimulus during natural infection.

We measured immune markers in the plasma and compared the change from baseline to immune marker concentrations at week 12 and 18, which corresponded to the average peak ALT. We observed a significant increase in sCD163, a monocyte/macrophage activation marker, at week 12 and 18. The significant increase in sCD163 was detectable in both men and women at week 12 but the magnitude of sCD163 increase was significantly greater in men and persisted until week 18 after stopping therapy (**Figure 1E**). Furthermore, we only observed significant increases in IL-6, IL-1α, TNF-α, CXCL-9, CCL4 and Fas ligand in men after stopping therapy (**Figure 1E**). CXCL-10 showed significant increases in both men and women at week 12 but, again, the magnitude of CXCL-10 was greater in men and remained significantly elevated at 18 weeks post stopping therapy (**Figure 1E**). Finally, we found that compared to females, men produced more sCD163, IL-6, IL-1α, TNF-α, and CCL4 at week 12. These data show that a liver-specific viral infection induces an enhanced myeloid inflammatory signature in men compared to women.

### The Intrasinusoidal Myeloid Composition Does Not Differ by Sex, Age, or BMI

**Figure 2:**
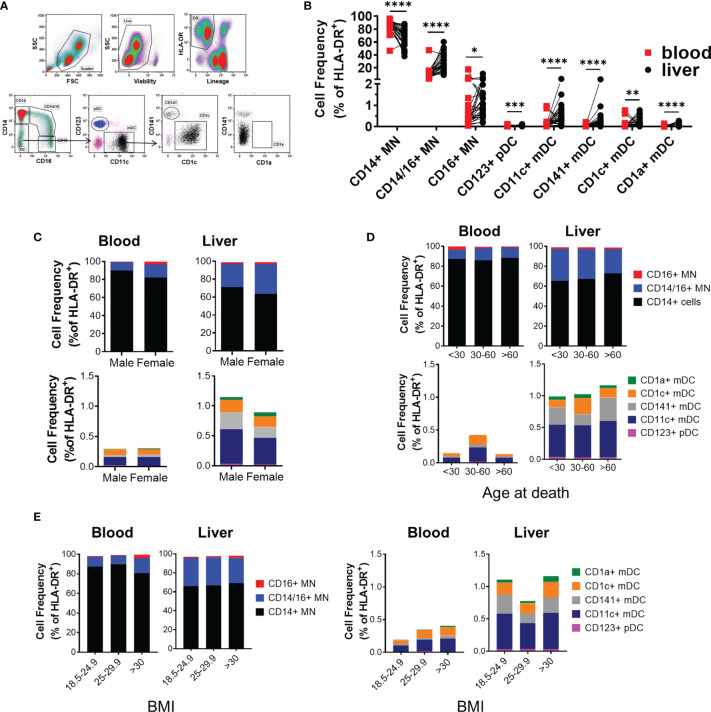
Intrahepatic mononuclear cells from liver perfusions display a profile of organ-specific immune cells. **(A)** Flow cytometry panel to measure the frequency of IHMC and matched PBMC myeloid frequencies. **(B)** Summarized myeloid cell frequencies between blood and liver (blood n=27, liver n = 30). Data on frequency displayed as percent of HLA-DR+ cells. Percent of total HLA-DR+ cells for each myeloid population based on **(C)** Sex (n=18 males, 12 females), **(D)** Age (<30, n= 8; 30-60, n=15; >60, n=7), or **(E)** BMI (18.5-24.9 n=13; 25-29.9 n=8; >30 n=9). *p < 0.05, **p < 0.01, ***p < 0.001, ****p < 0.0001, Statistical analyses: Mann-Whitney U tests and Kruskal-Wallis tests followed by Dunn’s multiple comparisons test when comparing cell frequencies by sex and across age groups.

Having verified the predisposition for men in developing hepatitis and defining a link to biomarkers of myeloid activation in active hepatitis in hepatitis B patients, we next investigated if sex impacts human liver immune composition. To study the immune composition of the liver, we used portal vein liver perfusions collected prior to transplant. Liver perfusions flush non-adherent, intrasinusoidal immune cells from the liver and have become a recognized source to obtain immune cells from uninfected liver transplant donors (16–19). We analyzed the frequency of seven intrasinusoidal and peripheral blood myeloid populations from liver donors using multiparametric flow cytometry (**Figure 2A**). Because neutrophils and other granulocytes are lost during density centrifugation and do not survive cryopreservation, these populations have been excluded from our analysis. In addition, we did not find a significant frequency of Kupffer cells in the perfusions using cytospin or MARCO and TIMD4 staining by flow cytometry (not shown). Kupffer cells are tightly bound to liver endothelial cells and likely require tissue digestion for mobilization (20).

The majority of myeloid cells in the intrahepatic mononuclear cells (IHMC) were classical CD14+ monocytes. However, classical CD14 monocytes (CD14 MN) were significantly reduced in the IHMC compared to matched PBMC (67.2 ± 2.6% vs. 86.2 ± 1.9% of HLA-DR+ myeloid cells respectively). In comparison, the liver contained a significantly higher proportion of CD14+CD16+ intermediate monocytes compared to the blood (28.69% and 11.23% of HLA-DR+ myeloid cells respectively). CD16+ monocytes made up 1-2% of HLA-DR+ myeloid cells in both compartments (**Figure 2B**). We similarly observed significant differences in the frequency of plasmacytoid DCs between the PBMC and IHMC. The majority of dendritic cells (DC) in the liver were CD11c myeloid derived DC. Conventional dendritic cell populations were enriched in the liver when compared to matched blood (**Figure 2B** and **Supplementary Table 4**). However, CD1a+ DC were extremely rare within liver perfusions as they have been described as tissue DCs and, similar to macrophages, likely require tissue digestion for full mobilization (21). As stated above, we did not find evidence for liver macrophages in the liver perfusion. These data demonstrate that myeloid frequencies in the liver differ significantly from blood and displayed a composition consistent with the intrahepatic environment.

We then compared the frequency of myeloid populations between male and female donors. Sex had no influence on myeloid composition (**Figure 2C** and **Supplementary Table 5**). Age is associated with inflammation (22). Therefore, we compared myeloid composition by age and, similarly, found that the intrahepatic myeloid composition did not change with age (**Figure 2D** and **Supplementary Table 6**). Similarly, when the effect of BMI on myeloid population distribution was investigated, we did not identify any relationship at healthy, overweight, and obese BMI with any population (**Figure 2E** and **Supplementary Table 7**). Overall, our data indicated that biological sex, age, and BMI had a minimal impact on myeloid composition, suggesting that the functional profile of myeloid cells may be responsible for the sex-bias in liver disease progression.

### TLR-Induced Cytokine Production Is Increased in Monocytes Isolated From Male Livers

**Figure 3:**
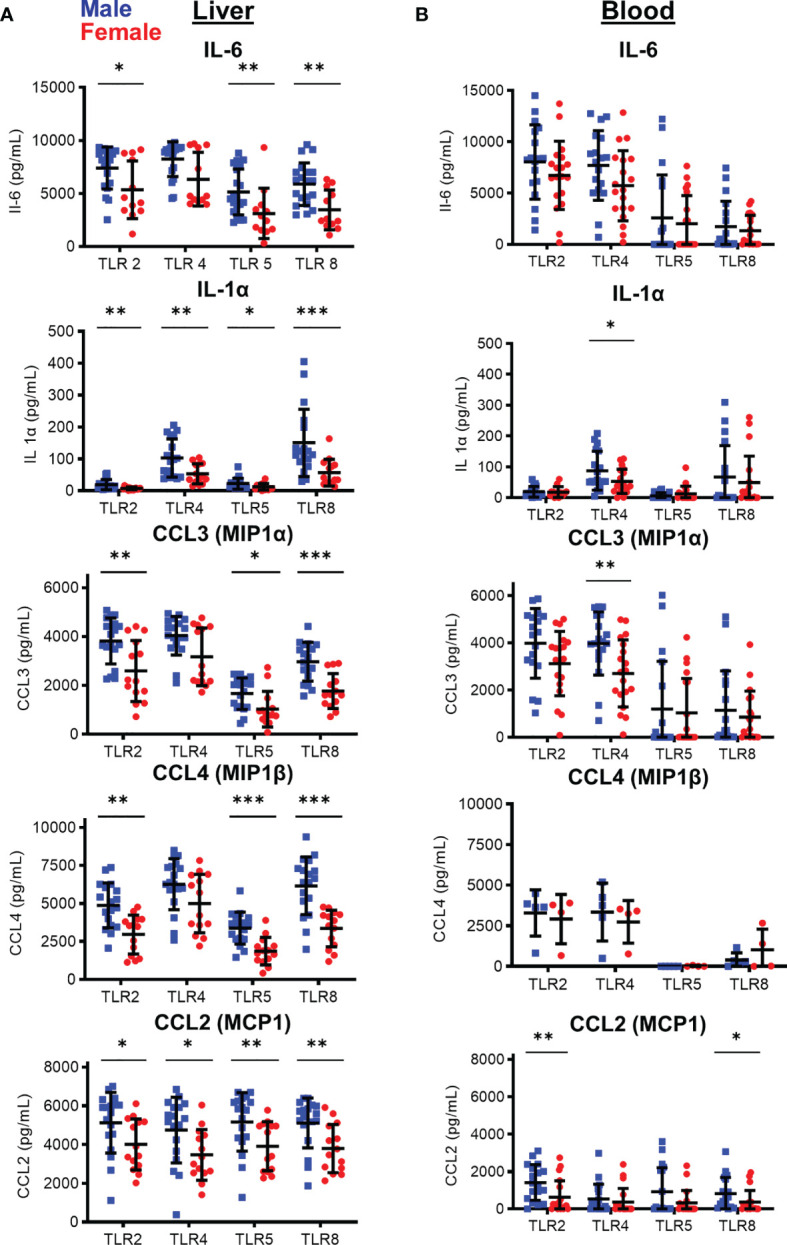
Sex-based differences in cytokine and chemokine production by monocytes following *in vitro* TLR stimulation of enriched CD14^+^ monocytes after 24 hours. Cytokine/chemokine production by **(A)** Liver and **(B)** peripheral blood isolated CD14+ monocytes after stimulation with TLR agonists. (IHMC males, n=18; females=14; PBMC: males, n=18; females, n=19; For CCL4 PBMC males, N=5; females=4) *p < 0.05, **p < 0.01, ***p < 0.001, Mann-Whitney U tests. Figures depict mean +/- SD.

With no differences in myeloid composition, we tested if the inflammatory potential of intrasinusoidal myeloid cells differed between males and females, as myeloid cells play key roles in liver inflammation and differentiate into inflammatory macrophages (11, 12). To investigate this, we tested the response of monocytes to inflammatory stimuli. Isolated monocytes were activated with agonists specific for TLR2, TLR3, TLR4, TLR5, and TLR8 for 24 h. After stimulation, we measured the production of 12 cytokines and chemokines (IL-1α, IL-1β, IL-6, IL-10, IL-12p70, CXCL-10, G-CSF, GM-CSF, MCP-1 (CCL2), Mip-1α (CCL3), Mip-1β (CCL4), and TNF-α). Intrasinusoidal monocytes robustly produced cytokines and chemokines in response to stimulation *via* TLR2, TLR4, TLR5, and TLR8 (**Figure 3A**). TLR3 activation was the only condition that induced CXCL-10 production, although overall responses to TLR3 were low in monocytes (data not shown). We also tested the response of blood-derived monocytes to TLR agonist stimulation (**Figure 3B**).

We observed significant differences in cytokine/chemokine production between male and female liver monocytes after stimulation of TLR2, TLR4, TLR5, and TLR8. The immune markers showing the most significant differences were IL-6 and IL-1α and the chemokines CCL3, CCL4, and CCL2 (**Figure 3A**). TNF-α, IL-10, IL-1β, and G-CSF also showed significant differences between men and women with select TLR stimulation (**Supplementary Figure 1**). CXCL-10, GM-CSF, and IL-12p70 did not show significant differences between men and women (**Supplementary Figure 1**). We did not find a significant correlation between spontaneous cytokine production and alanine aminotransferase (ALT) levels, the clinical marker of liver damage, confirming the differences between men and women were not due to liver damage at the time of organ transplant (**Supplementary Figure 2**). The most significant differences in cytokine/chemokine production were consistently observed after stimulation of TLR8, followed by TLR5, TLR2, and TLR4.

To determine if this differential response between male and female monocytes was restricted to the liver, we isolated monocytes from the peripheral blood of men and women and compared cytokine and chemokine production. In contrast to the intrasinusoidal data, significant difference between blood monocytes from men and women were much more restricted, with only IL-1α and CCL3 showing significant difference after TLR4 stimulation, and CCL2 after TLR2 and TLR8 stimulation (**Figure 3B**). All other responses did not show a significant difference between men and women in blood monocytes (**Figure 3B** and **Supplementary Figure 1**). These data show that, while statistically significant functional differences are detectable in the blood, functional differences between male and female monocytes are magnified in the liver and male liver monocytes respond to inflammatory stimuli with enhanced cytokine and chemokine production.

### No Phenotypic Difference in Intrasinusoidal Monocytes Between Men and Women

**Figure 4:**
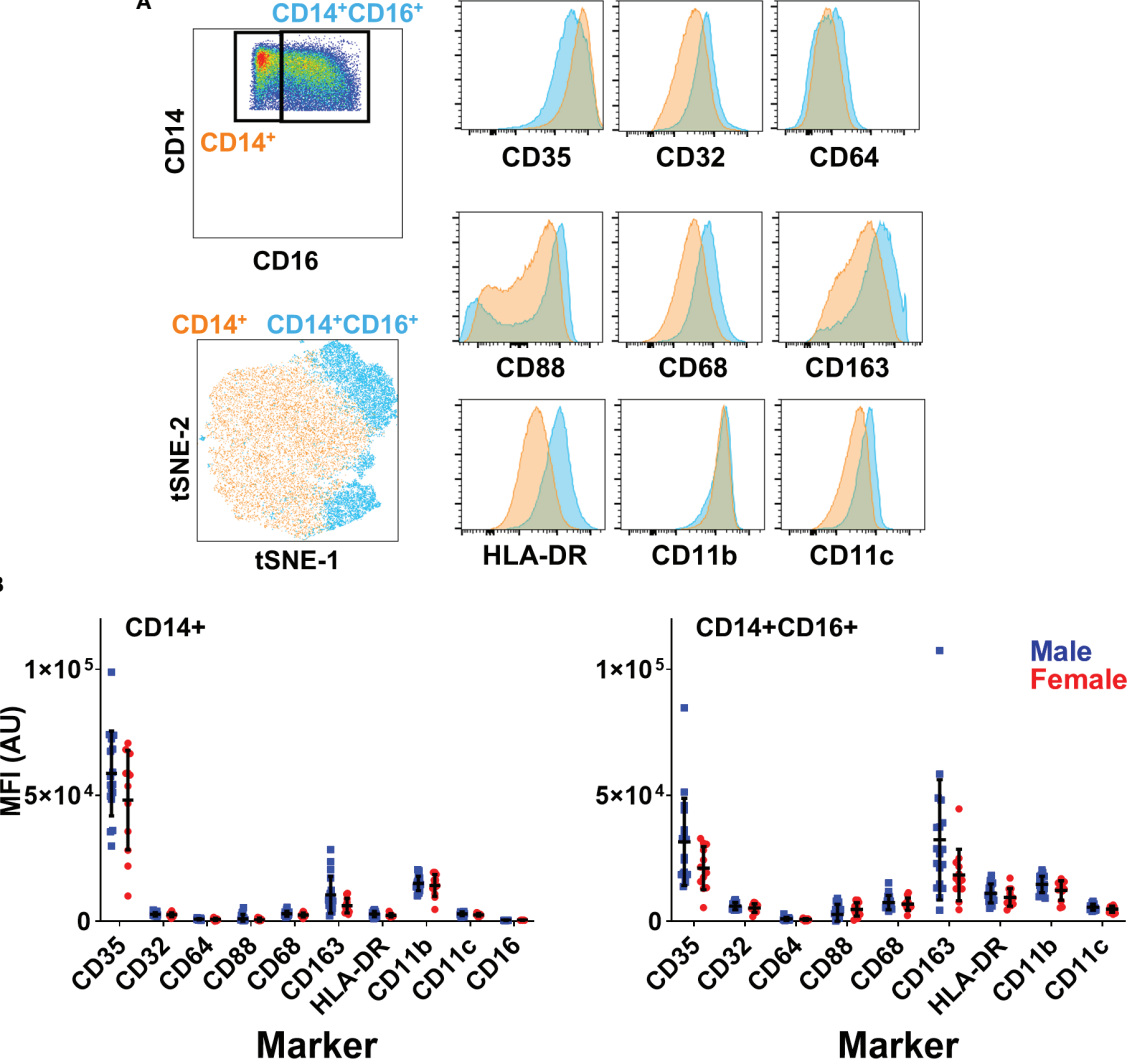
No differences in monocyte phenotype based on sex. **(A)** Surface phenotype of liver CD14+ and CD14+CD16+ monocytes. T-SNE dimensionality reduction was performed on monocytes concatenated from all liver samples (5000 cells per sample). **(B)** Expression of surface markers in monocyte subsets by sex n=18 males, 12 females. Figures depict mean +/- SD.

With no difference observed in monocyte frequency, but a significant difference in function, we sought to determine if the liver environment altered monocyte phenotype between men and women, potentially enhancing their response to activating signals. Therefore, we performed deeper phenotypic analysis on monocytes, measuring the expression of scavenger receptors (CD68, CD163), complement receptors (CD35, CD88), Fc receptors (CD16, CD32, CD64) and integrins (CD11c, CD11b) on CD14+ MN and CD14/CD16+ MN (**Figure 4A**). We did not observe any significant phenotypic differences between monocytes from male and female livers (**Figure 4B**). This data indicates that neither the composition nor phenotype of monocytes differs significantly by sex.

### Regulation of TLR-Induced Cytokine Production

**Figure 5:**
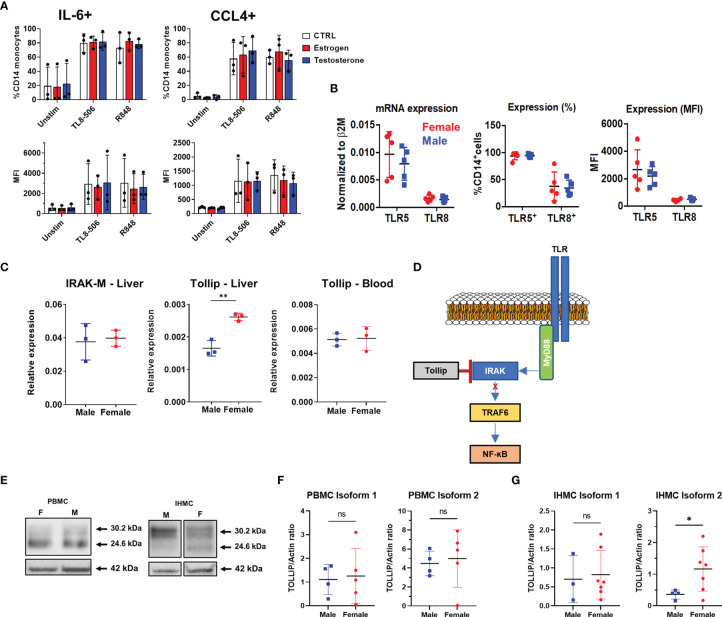
Investigation of TLR expression and signaling. **(A)** Total HD PBMCs from exposed to estrogen or testosterone for 18 hours were stimulated with TLR8 agonists in the presence of Brefeldin A and IL-6 and CCL-4 production in CD14+ MN were measured by flow cytometry. Data displayed as percent of monocyte positive for each cytokine(top) or MFI (bottom) n=3 PBMC donors. **(B)** TLR expression in male and female liver monocytes. mRNA measured by qPCR (left) and protein expression measured by flow cytometry (right) n=5 males, 5 females. **(C)** Analysis of negative regulators of TLR signaling. TOLLIP expression was measured in both the liver (left) and blood (right). PBMC n=5 male, 5 female; IHMC n=3 male, 3 female. **(D)** Representation of TOLLIP’s intersection of the TLR signaling cascade to demonstrate the potential to influence all TLRs. **(E)** Example western blot of TOLLIP protein (top) and Actin (bottom) in purified CD14+ monocytes. **(F)** TOLLIP isoform expression in blood monocytes does not show a significant relationship between sex and the expression of TOLLIP. **(G)** TOLLIP isoform 2 expression is elevated in female liver monocytes. Densitometry data was subject to outlier analysis with Grubb’s method. PBMC n=5 male, 5 female; IHMC n=4 males, 8 female, *p<0.05, **p<0.01, ns, non-significant, Mann-Whitney U tests. Figures depict mean +/- SD.

With no difference in frequency or phenotype of male liver monocytes to explain heightened inflammatory responses, we next investigated whether sex hormones, genetic expression, or the TLR signaling cascade showed differences between men and women. We focused our mechanistic analysis on TLR8 and TLR5 as these TLRs displayed the most significant functional differences between men and women. In addition, TLR8 is located on the X chromosome while TLR5 is not, allowing us to compare whether genetic expression plays a role. We first tested the effect of sex hormone exposure on TLR8-induced IL-6 and CCL4 production. Pre-incubation with either estrogen or testosterone had no impact on the frequency, or staining intensity, of IL-6 or CCL4 in CD14+ MN compared to untreated cells (**Figure 5A**). We then investigated whether TLR5 or TLR8 expression differed, at both the mRNA or protein level. We found no difference between male and female liver monocytes in terms of TLR5 and TLR8 mRNA expression, the percent of liver monocytes expressing the TLRs or the level of TLR expression based on mean fluorescence intensity (**Figure 5B**). Therefore, neither sex hormone exposure nor TLR expression could explain differences in cytokine/chemokine production between male and female liver monocytes.

Because we observed reduced cytokine and chemokine production in *ex vivo* stimulated monocytes from female livers, we chose to investigate negative regulators of TLR signaling. Specifically, we investigated the expression of both IRAK-M and TOLLIP. The expression of IRAK-M did not differ by sex. However, we observed that female liver monocytes expressed more TOLLIP when compared to male liver monocytes. When we investigated the expression of TOLLIP in blood monocytes, we did not find that sex influenced TOLLIP expression (**Figure 5C**). We also investigated expression of key molecules in the TLR signaling cascade and terminal transcription factors for TLRs signaling, including MyD88, IRAK-4, IRAK-1, TRAF-6, RelA (NF-ĸB), and IRF-7 (**Supplementary Figures 3A, B**), but found no statistically significant differences between male and female liver monocytes. These data suggest liver-restricted regulation of TOLLIP, with increased expression of the negative regulator of TLR signaling in female liver monocytes.

TOLLIP regulates IRAK phosphorylation and is known to suppress TLR-mediated inflammatory cytokine production in human monocytes [**Figure 5D** (23)]. To determine if differences in mRNA expression translated to differences in TOLLIP protein expression, we used Western blot to confirm TOLLIP protein expression in liver monocytes from donors who underwent living donor liver transplants. Western blot data identified two known isoforms (isoform 1 and isoform 2; 30.2 and 24.6 kDa, respectively) of TOLLIP protein in both male and female monocytes (**Figure 5E** and **Supplementary Figures 4A, B**). Comparison of the expression of TOLLIP isoform 1 and 2 in monocytes from 10 PBMC donors revealed no statistically significant difference in expression between males and females (**Figure 5F**). Quantification of TOLLIP expression in liver monocytes revealed that while TOLLIP isoform 1 expression was similar between males and females, we found that the expression of TOLLIP isoform 2 was statistically significantly elevated in female liver monocytes (**Figure 5G**). These data suggest that the MyD88 signaling cascade may be differentially regulated between the blood and liver and between men and women within the liver. 

## Discussion

It is generally accepted that females display stronger cell mediated immune responses than males. Females have increased CD4 T cells (24), show stronger responses to vaccination (25–27) and have higher incidence of autoimmune disorders mediated by cells of the adaptive immune system (28). However, the inflammatory potential of innate immunity between men and women is less clearly defined and may be restricted to specific cell types or specific receptors. An extensive study performed by Human Functional Genomics Project (HGFP) found that monocyte-derived cytokines were higher in men vs. women (29). This study stimulated whole blood and PBMC from 500 healthy volunteers and found higher IL-6, IL-1β and TNF-α in males. The study was of low resolution in identifying relevant cell populations as they used whole blood and PBMC, instead of purified monocytes. Further, this study required a large patient cohort to demonstrate differences but are consistent with our data on purified monocytes. Similarly, a recent study also supported the observation of enhanced inflammatory potential of peripheral blood male monocytes, but analysis was focused on TNF-α, CXCL1, and CCL2 (30). Our data indicates that differences in cytokine production between male and female monocytes was magnified in the liver, suggesting regulation, at least in part, by a tissue-specific effect.

We first validated the clinical observations that men have worsened outcomes in hepatitis through a clinical cohort of patients with chronic HBV infection going through treatment withdrawal. We found that sCD163 was the most statistically significantly elevated marker in patients with HBV reactivation. Importantly, CD163 is expressed on monocytes and macrophages and is shed from their surface upon TLR activation (31, 32). Further, previous work has correlated sCD163 with HBV-related liver inflammation (33, 34). The increased inflammatory profile in male patient plasma was consistent with subsequent TLR activation on monocytes and macrophages. The myeloid cytokine/chemokine profile we observed in CHB patients matched closely with our *ex vivo* liver monocyte data, with higher IL-6, IL-1α, CCL-4, and TNF-α observed in male donors. Plus, additional chemokines, CXCL-9 and CXCL-10, were higher in men and have been associated with liver inflammation (35, 36). It is possible that the inflammatory cytokine profile we observed in CHB patients was not produced by liver-resident monocytes/macrophages, but this scenario is unlikely. HBV virions and antigens constantly circulate through the peripheral blood at high levels for the lifetime of the patients and do not engage innate inflammatory mechanisms (15, 37). In contrast, HBV reactivation triggers a poorly understood liver-specific danger signal, which induces liver inflammation in a majority of patients stopping antiviral therapy (38, 39). Therefore, we believe the immune profile we detected in the CHB patient cohort is derived from the liver and is indicative of monocyte activation. While we were unable to investigate the relationship between different myeloid populations in the blood and liver in this study cohort, it has been reported that HBV DNA and HBV antigens do not influence the distribution of myeloid population in blood (40, 41). However, investigating dynamic changes in these immune populations during active hepatitis may be key to understanding population and phenotype kinetics in *in vivo* inflammation.

Identification of specific mechanisms regulating sex-biased immune responses have been challenging due to the broad effects of genetics, environment and hormones (42). Sex hormone concentrations fluctuate and regulate the promoters of multiple immune-related genes. The X chromosome harbors numerous immune genes, including TLR7 and TLR8, and microRNAs with reported effects on immune function (43). We focused our mechanistic studies on TLR8 and TLR5, which allowed us to compare hormone and X chromosome dependent effects on protein expression. Our observation, that cytokine and chemokine production were similar in peripheral blood monocytes between men and women argues against direct regulation by sex hormones, as these hormones are systemic. Our *in vitro* mechanistic data involving sex-hormone exposure supported this conclusion, which was also found in the HFGP cohort (29). Similarly, our data did not show differences in mRNA or protein expression between TLR5 and TLR8, arguing against increased expression from the X chromosome.

Finding a significant difference in cytokine production after stimulation with all TLR agonists suggested a common mechanism in the signaling cascade. This would be similar to the regulation of IFN-α production after TLR7 stimulation in female plasmacytoid DC, where the terminal transcription factor, IRF-5 was increased (44). Our interrogation of the signaling cascade determined that the negative regulator of TLR signaling, TOLLIP, was expressed at higher levels in female liver monocytes compared to male monocytes, with no difference in the blood, suggesting liver-specific regulation of the TOLLIP promoter. TOLLIP interacts with the IRAK complex to reduce downstream phosphorylation events, reducing cytokine production in human monocytes (23, 45, 46). Recent studies have demonstrated that transduction of primary human monocytes with short hairpin RNA to knockdown TOLLIP enhanced cytokine production following TLR stimulation (23). Similarly, overexpression of TOLLIP in murine monocytes could dramatically suppress TLR signaling through NF-ĸB dependent pathways (47). Our Western blot data confirmed TOLLIP protein expression in both blood and liver monocytes from men and women. With our limited sample size for protein analysis, we observed a statistically significant increase in TOLLIP isoform 2 in female liver monocytes. The intersection of TOLLIP with the TLR signaling cascade is consistent with our functional data demonstrating that TLR2, -5, and -8, which signal through MyD88-IRAK, showed the most significant differences. This data is also consistent with the key role of MyD88-dependent IL-6 production in the development of liver cancer in male mice (48). In contrast, TLR4 signaling can bypass MyD88-IRAK through the TRIF pathway and showed the fewest significant differences between male and female liver monocytes. Identification of key factors that can dampen inflammatory cytokine production in the human liver may yield targets to treat, and slow the progression of, multiple types of inflammatory liver disease.

We recognize there are caveats to our uninfected liver analysis. Liver perfusion samples are collected from deceased donors. All the livers used in our analysis were used for transplantation, verifying the quality of the organs. The cause of death or age at death could skew results. However, cause of death was distributed among all categories for both men and women. Age at death was not significantly different and *ex vivo* cytokine production did not correlate with the donor’s APRI score (not shown) or ALT levels. Therefore, we are confident the differences we observed are physiologically accurate and not a consequence of the liver donors. Additionally, the cells available for analysis from the perfusion samples are those that can be flushed from the sinusoid upon portal vein perfusion. These were highly representative of liver derived cells but also do not capture tightly bound macrophages or parenchymal cells. Until the data are obtained, whether macrophages or parenchymal cells respond with heightened inflammatory potential remains an open question.

Our study characterizes the human liver, between sexes, in the absence of underlying liver disease. This data is vitally important to understanding the mechanisms driving liver inflammation that put hundreds of millions of individuals at risk of developing cirrhosis and liver cancer. We believe the sex-bias inflammatory potential is a general phenomenon of the intrasinusoidal environment. Elucidating liver-specific regulation of cytokine production between men and women, will further improve efforts to manage the pathogenesis of liver disease.

## Data Availability Statement

The original contributions presented in the study are included in the article/**Supplementary Material**. Further inquiries can be directed to the corresponding author.

## Ethics Statement

The studies involving human participants were reviewed and approved by Saint Louis University institutional review board and the University Health Network research ethics board. Written informed consent to participate in this study was provided by the participants’ legal guardian/next of kin.

## Author Contributions

AK, DM, SN, JAD, AM, AJV acquired and analyzed data; AK, DM, SN, JAD, WC, PH, IM, HLAJ, AG were responsible for study design and manuscript preparation and revision. All authors contributed to the article and approved the submitted version.

## Funding

This study was funded by a grant from the Saint Louis University Liver Center institutional funding from Saint Louis University and the Toronto Centre for Liver Disease and a grant from the Gilead Research Scholars Program in Liver Disease. The funder was not involved in the study design, collection, analysis, interpretation of data, the writing of this article or the decision to submit it for publication.

## Conflict of Interest

The authors declare that the research was conducted in the absence of any commercial or financial relationships that could be construed as a potential conflict of interest.

## Publisher’s Note

All claims expressed in this article are solely those of the authors and do not necessarily represent those of their affiliated organizations, or those of the publisher, the editors and the reviewers. Any product that may be evaluated in this article, or claim that may be made by its manufacturer, is not guaranteed or endorsed by the publisher.
