# Supplementary figures and images for: The Human Male Liver Is Predisposed to Inflammation Via Enhanced Myeloid Responses to Inflammatory Triggers
Source: Front Immunol. 2022 Apr 14;13:818612. doi: 10.3389/fimmu.2022.818612 (PMC9046993; doi:10.3389/fimmu.2022.818612)

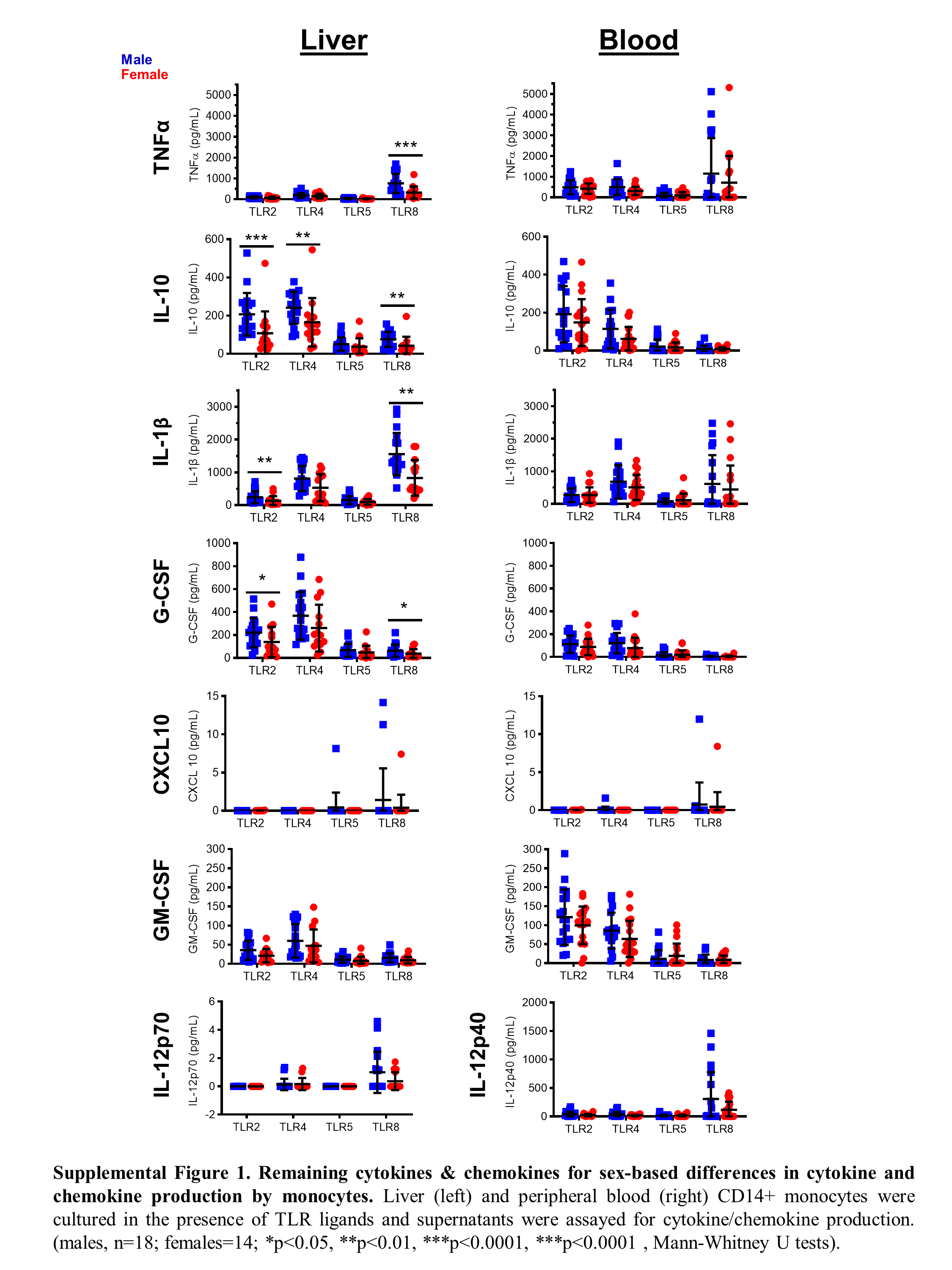

Supplement: Supplementary file 1 [file Image_1.tif]

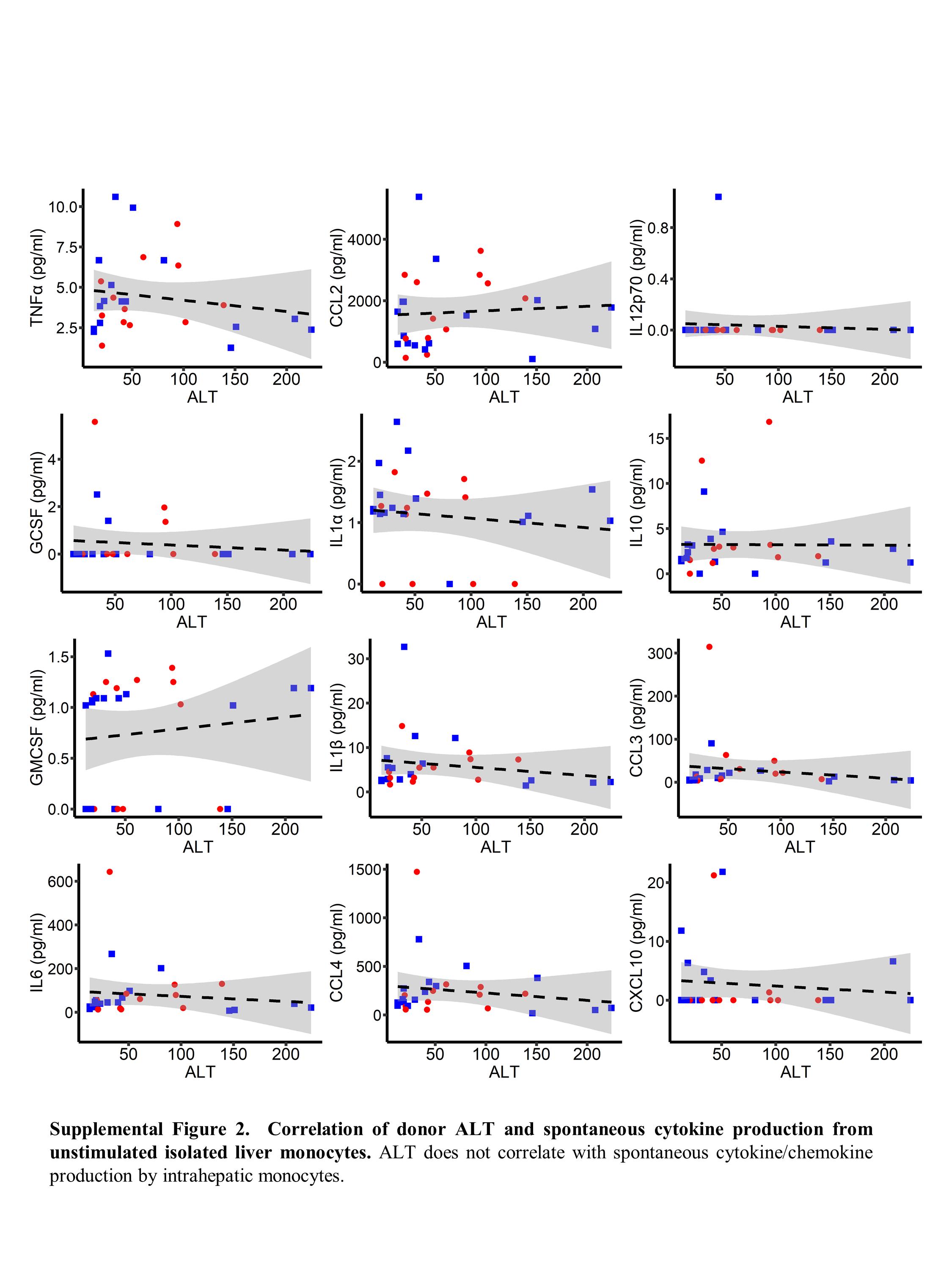

Supplement: Supplementary file 2 [file Image_2.tif]

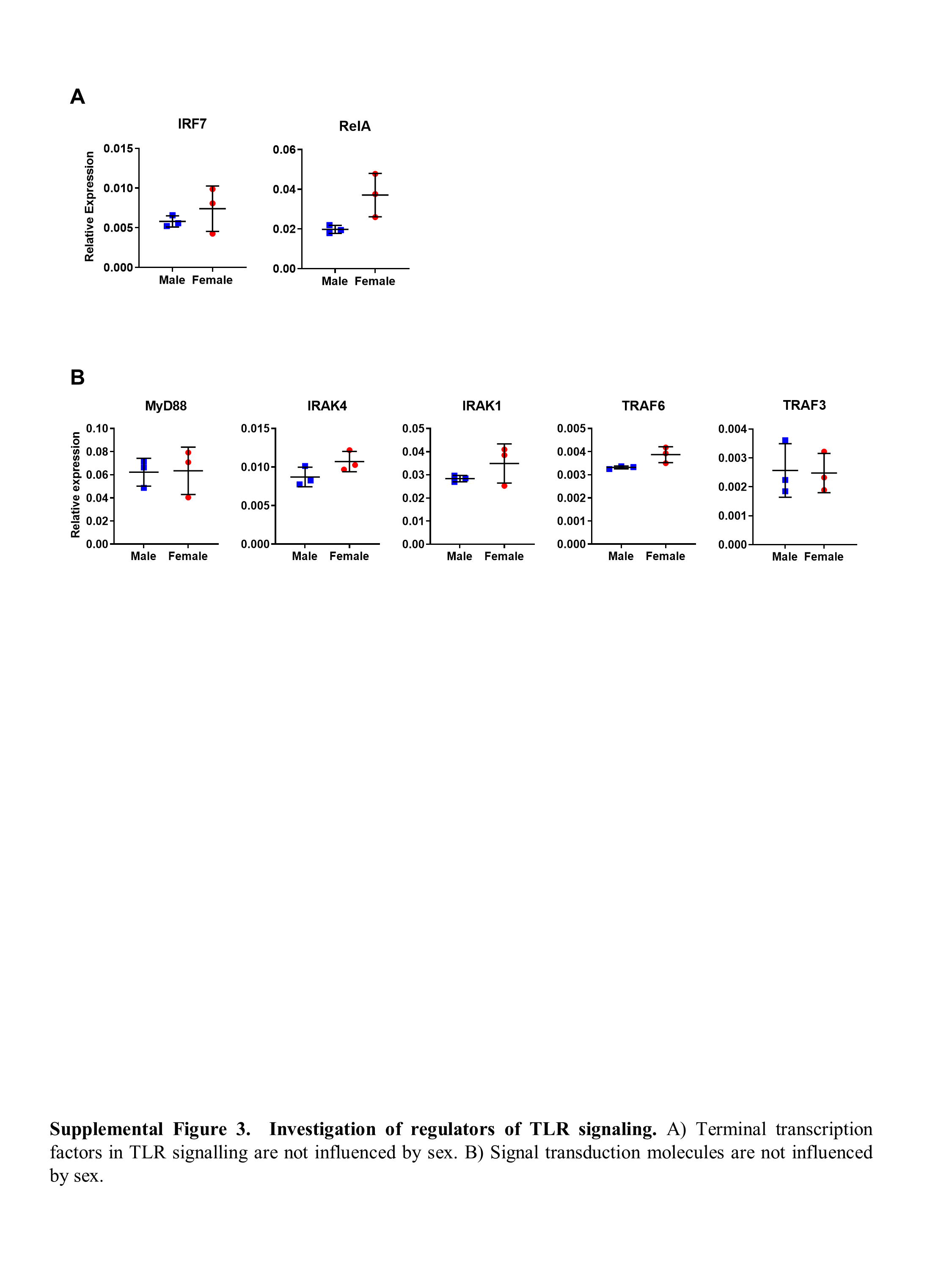

Supplement: Supplementary file 3 [file Image_3.tif]

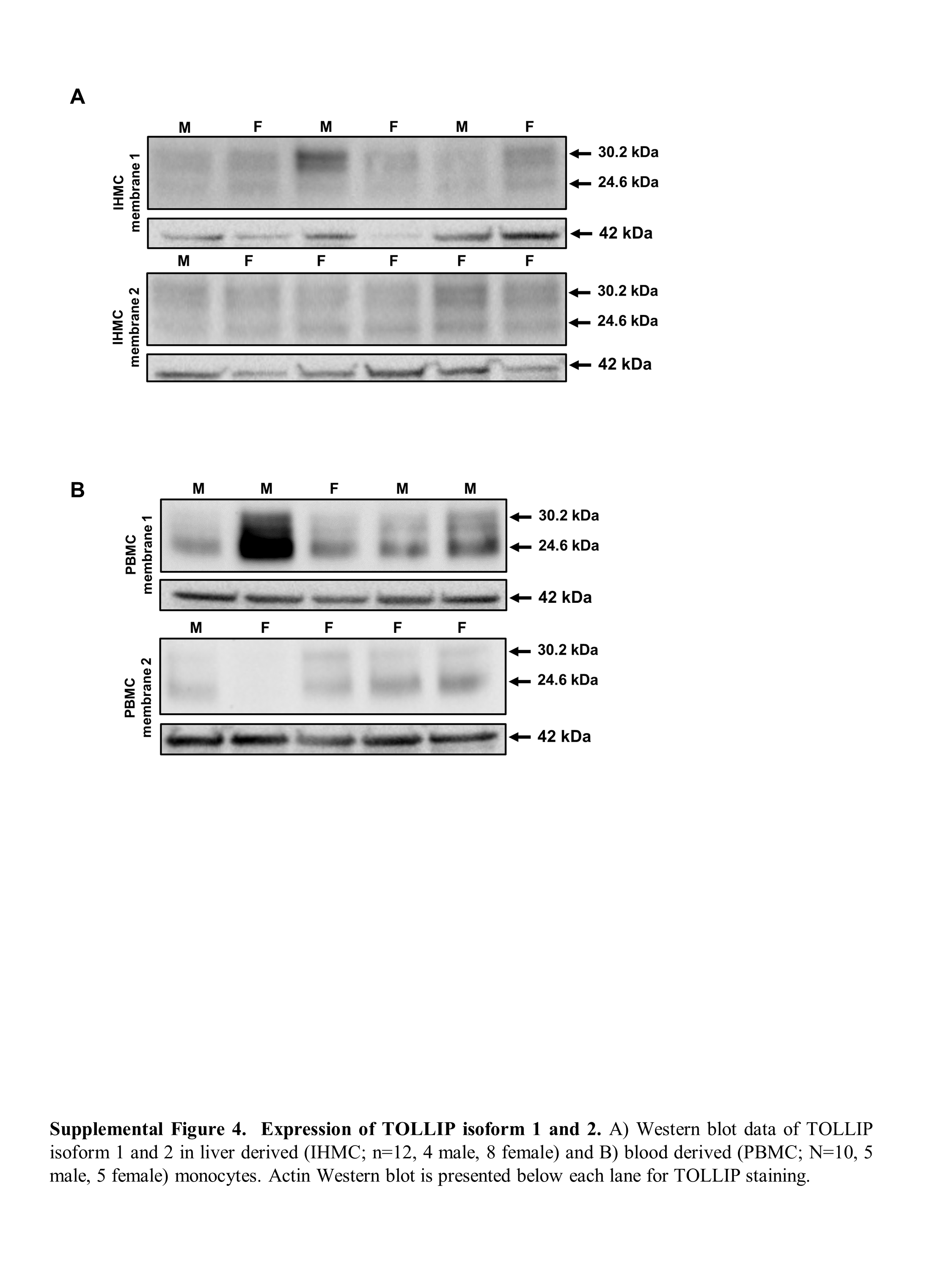

Supplement: Supplementary file 4 [file Image_4.tif]
