# Supplementary material for: The Human Male Liver Is Predisposed to Inflammation Via Enhanced Myeloid Responses to Inflammatory Triggers
Source: Front Immunol. 2022 Apr 14;13:818612. doi: 10.3389/fimmu.2022.818612 (PMC9046993; doi:10.3389/fimmu.2022.818612)
Supplement: Supplementary file 5 [file Table_1.pdf]

Supplemental TABLES

Table S1. Characteristics of healthy liver perfusions

Characteristic	Female	Male
Number	21	32
Race (W/AA/I[1])	17/4/0	22/9/1
Median age at death	42 (18-73)	35 (10-68)
Median BMI*	31.3(21.1-70.7)	25.7(19.2-47.7)
ALT	42(14-139)	45(13-306)

[1]White/African American/Indian
*p<0.05

Table S2. Reported mechanisms of death

Mechanism of Death	Male	Female
Intracranial hemorrhage/stroke	9(28%)	7(33%)
Blunt injury	8(25%)	1(5%)
Cardiovascular	5(16%)	5(24%)
Gunshot wound	4(13%)	1(5%)
Asphyxiation	2(6%)	1(5%)
Death from natural causes	2(6%)	3(14%)
Drug/intoxication	2(6%)	2(10%)
Unreported	0(0%)	1(5%)

Table S3. Distribution of myeloid cell populations in blood and liver

**Myeloid population**	**Blood**	**Liver**
CD14+ MN	86.198 (10.018)	67.152 (14.374)
CD14/16+ MN	11.228 (8.078)	28.690 (13.003)
CD16+ MN	1.489 (3.424)	1.688 (2.157)
CD123+ pDC	0.013 (0.024)	0.031 (0.031)
CD11c+ mDC	0.147 (0.229)	0.515 (0.56)
CD141+ mDC	0.033 (0.043)	0.240 (0.394)
CD1c+ mDC	0.094 (0.117)	0.193 (0.206)
CD1a+ mDC	0.011 (0.028)	0.055 (0.074)

Mean % (SD)

Table S4. Distribution of myeloid cell populations in blood and liver by sex

	**Blood**		**Liver**	
**Myeloid population**	**Male**	**Female**	**Male**	**Female**
CD14+ MN	89.88 (4.3)	80.84 (13.4)	70.72 (13.5)	61.8 (14.5)
CD14/16+ MN	9.03 (4.2)	14.42 (11.1)	26.16 (12.1)	32.49 (13.9)
CD16+ MN	0.58 (0.7)	2.81 (5.1)	1.38 (1.3)	2.15 (3)
CD123+ pDC	0.017 (0.03)	0.0063 (0.01)	0.034 (0.04)	0.025 (0.02)
CD11c+ mDC	0.14 (0.2)	0.15 (0.3)	0.57 (0.6)	0.43 (0.4)
CD141+ mDC	0.028 (0.02)	0.04 (0.06)	0.28 (0.5)	0.18 (0.3)
CD1c+ mDC	0.098 (0.2)	0.09 (0.2)	0.21 (0.2)	0.17 (0.2)
CD1a+ mDC	0.0088 (0.01)	0.014 (0.04)	0.046 (0.06)	0.068 (0.09)

Mean (SD)

Table S5. Distribution of liver myeloid cell populations by age group.

	Blood			Liver		
**Myeloid population**	<30	30-60	>60	<30	30-60	>60
CD14+ MN	86.97 (8.1)	85.83 (12.5)	86.22 (7.4)	64.1 (11.7)	67.72 (16.2)	69.22 (14.6)
CD14/16+ MN	9.31 (2.7)	12.38 (11.2)	10.75 (3.9)	31.56 (9.1)	28.77 (15.6)	25.68 (12.2)
CD16+ MN	3.21 (7)	1.16 (1.5)	0.73 (1)	1.46 (1.7)	1.9 (2.7)	1.54 (1.6)
CD123+ pDC	0.005 (0.005)	0.023 (0.03)	0.0037 (0.005)	0.034 (0.03)	0.033 (0.03)	0.024 (0.04)
CD11c+ mDC	0.077 (0.06)	0.23 (0.3)	0.071 (0.05)	0.5 (0.4)	0.53 (0.3)	0.5 (0.9)
CD141+ mDC	0.018 (0.02)	0.046 (0.05)	0.021 (0.02)	0.27 (0.3)	0.18 (0.1)	0.31 (0.7)
CD1c+ mDC	0.047 (0.04)	0.15 (0.2)	0.029 (0.02)	0.11 (0.09)	0.27 (0.2)	0.13 (0.2)
CD1a+ mDC	0 (0)	0.02 (0.04)	0.0037 (0.007)	0.055 (0.08)	0.064 (0.7)	0.037 (0.7)

Mean (SD)

Table S6. Distribution of myeloid cell populations in blood and liver by BMI.

	Blood			Liver		
**Myeloid population**	**18.5-24.9**	**25-29.9**	**>30**	**18.5-24.9**	**25-29.9**	**>30**
CD14+ MN	87.49 (7)	90.11 (3.2)	80.84 (15.4)	65.98 (16.3)	66.99 (9.8)	68.99 (16.2)
CD14/16+ MN	9.96 (4.3)	8.79 (3.7)	15.26 (13.2)	29.77 (15.6)	29.3 (8.1)	26.58 (13.5)
CD16+ MN	0.86 (1.1)	0.4 (0.3)	3.39 (6)	1.22 (1.4)	1.47 (1.1)	2.55 (3.4)
CD123+ pDC	0.012 (0.01)	0.021 (0.04)	0.0075 (0.1)	0.028 (0.03)	0.036 (0.04)	0.029 (0.02)
CD11c+ mDC	0.095 (0.06)	0.17 (0.3)	0.2 (0.3)	0.55 (0.7)	0.4 (0.3)	0.57 (0.5)
CD141+ mDC	0.028 (0.03)	0.024 (0.02)	0.046 (0.7)	0.3 (0.5)	0.15 (0.1)	0.24 (0.3)
CD1c+ mDC	0.051 (0.04)	0.12 (0.3)	0.13 (0.2)	0.18 (0.2)	0.16 (0.2)	0.24 (0.2)
CD1a+ mDC	0.0067 (0.01)	0.0071 (0.02)	0.02 (0.05)	0.045 (0.06)	0.034 (0.04)	0.088 (0.1)

Mean (SD)

Table S7. List of antibodies

Target	Source	Clone
CD1a	BD Biosciences	HI149
CD1c	Biolegend	L161
CD11b	BD Biosciences	ICRF44
CD11c	BD Biosciences	B-LY6
CD14	BD Biosciences	MphiP9
CD16	Biolegend	3G8
CD32	Biolegend	FUN-2
CD35	Biolegend	E11
CD45	BD Biosciences	HI30
CD64	BD Bioscience	10.1
CD68	BD Bioscience	Y1/82A
CD88	Biolegend	S5/1
CD123	BD Biosciences	7G3
CD141	Miltenyi	AD5-14H12
CD163	Biolegend	GHI/61
HLA-DR	Biolegend	L243
TRAIL	Biolegend	RIK-2
Tollip	Biolegend	O92A4
Actin	BD Bioscience	C4/actin
